# Surgical Robotics for Intracerebral Hemorrhage Treatment: State of the Art and Future Directions

**DOI:** 10.1007/s10439-023-03295-x

**Published:** 2023-07-05

**Authors:** Zhuojin Wu, Danyang Chen, Chao Pan, Ge Zhang, Shiling Chen, Jian Shi, Cai Meng, Xingwei Zhao, Bo Tao, Diansheng Chen, Wenjie Liu, Han Ding, Zhouping Tang

**Affiliations:** 1 Department of Neurology, Tongji Hospital, Tongji Medical College, Huazhong University of Science and Technology, Wuhan, 430030 China; 2 School of Mechanical Science & Engineering, Huazhong University of Science and Technology, Wuhan, 430074 China; 3 School of Mechanical Engineering & Automation-BUAA, Beihang University, Beijing, 100083 China; 4 Beijing WanTeFu Medical Instrument Co., Ltd, Beijing, 102299 China

**Keywords:** Hematoma evacuation, Intracerebral hemorrhage, Navigation, Perception, Puncture, Robot-assisted surgery

## Abstract

Intracerebral hemorrhage (ICH) is a stroke subtype with high mortality and disability, and there are no proven medical treatments that can improve the functional outcome of ICH patients. Robot-assisted neurosurgery is a significant advancement in the development of minimally invasive surgery for ICH. This review encompasses the latest advances and future directions of surgical robots for ICH. First, three robotic systems for neurosurgery applied to ICH are illustrated. Second, the key technologies of robot-assisted surgery for ICH are introduced in aspects of stereotactic technique and navigation, the puncture instrument, and hematoma evacuation. Finally, the limitations of current surgical robots are summarized, and the possible development direction is discussed, which is named “multisensor fusion and intelligent aspiration control of minimally invasive surgical robot for ICH”. It is expected that the new generation of surgical robots for ICH will facilitate quantitative, precise, individualized, standardized treatment strategies for ICH.

## Introduction

Intracerebral hemorrhage (ICH) refers to nontraumatic brain parenchymal hemorrhage, which is the second most detrimental subtype in stroke patients, with a mortality as high as 35%−52% [1]. Only approximately 20% of patients are able to achieve functional independence within 6 months after clinical treatment [2].

Acute medical management plays a crucial role in treating ICH by preventing its deterioration, which is achieved through measures such as controlling blood pressure to prevent hematoma expansion, and reversing anticoagulant effects if necessary. Additionally, it involves preventing and managing secondary brain injuries that may arise from seizures, elevated intracranial pressure, hyperglycemia, and fever [3]. Although various medical treatments have been explored, there is currently no specific treatment that has been proven to improve outcomes for ICH patients. Intuitively, hematoma removal could be a potentially effective therapy for ICH. The space-occupying effect and toxic content release are the main mechanisms by which hematoma leads to brain cell death. It has been suggested that the longer hematoma is present, the worse the prognosis is [4]. Thus, the key to the treatment is to remove hematoma as early as possible and prevent rebleeding [5]. However, studies have yet to demonstrate a significant functional outcome benefit from surgical intervention. The results of the STICH I and STICH II trials indicated that traditional craniotomy failed to reduce mortality or improve prognosis in ICH patients compared with conservative drug therapy [6, 7]. The limited efficiency of traditional craniotomy on ICH could be attributed to counterbalancing of the inevitable injury to normal brain tissue during surgical manipulation and the benefits of hematoma removal, which makes minimally invasive surgery (MIS) the most promising surgical strategy. MIS can reduce mechanical damage to the surrounding normal tissues and shorten the duration of surgery, which includes neuroendoscopic surgery and stereotactic hematoma puncture and drainage followed by thrombolysis [8, 9]. The MISTIE III trial showed that although MIS reduced all-cause mortality, it did not provide any functional outcome advantages in patients one year after ICH [10].

Robot-assisted surgery is a challenging and evolving technique, the application of which has been recently extended to certain surgical cases for treating ICH. Compared with traditional procedures, it has the advantages of high-accuracy position, short operative time, and considerable anti-interference, which could reduce the error of manual operation and, thus, enable a higher level of security. The meta-analysis by Xiong et al. suggested that robot-assisted MIS showed an overall superiority over conventional surgery or conservative management for ICH in terms of rebleeding rate, neurological function improvement and intracranial infection rate [11]. The era of precision medicine heralds much promise in developing more efficacious and personalized therapies to combat this disease. Therefore, robot-assisted surgery is considered a significant direction for the development of future minimally invasive strategies for ICH. This review encompasses the latest advances and future directions of surgical robots for ICH.

## Current Status of Surgical Robots for ICH

**Fig. 1:**
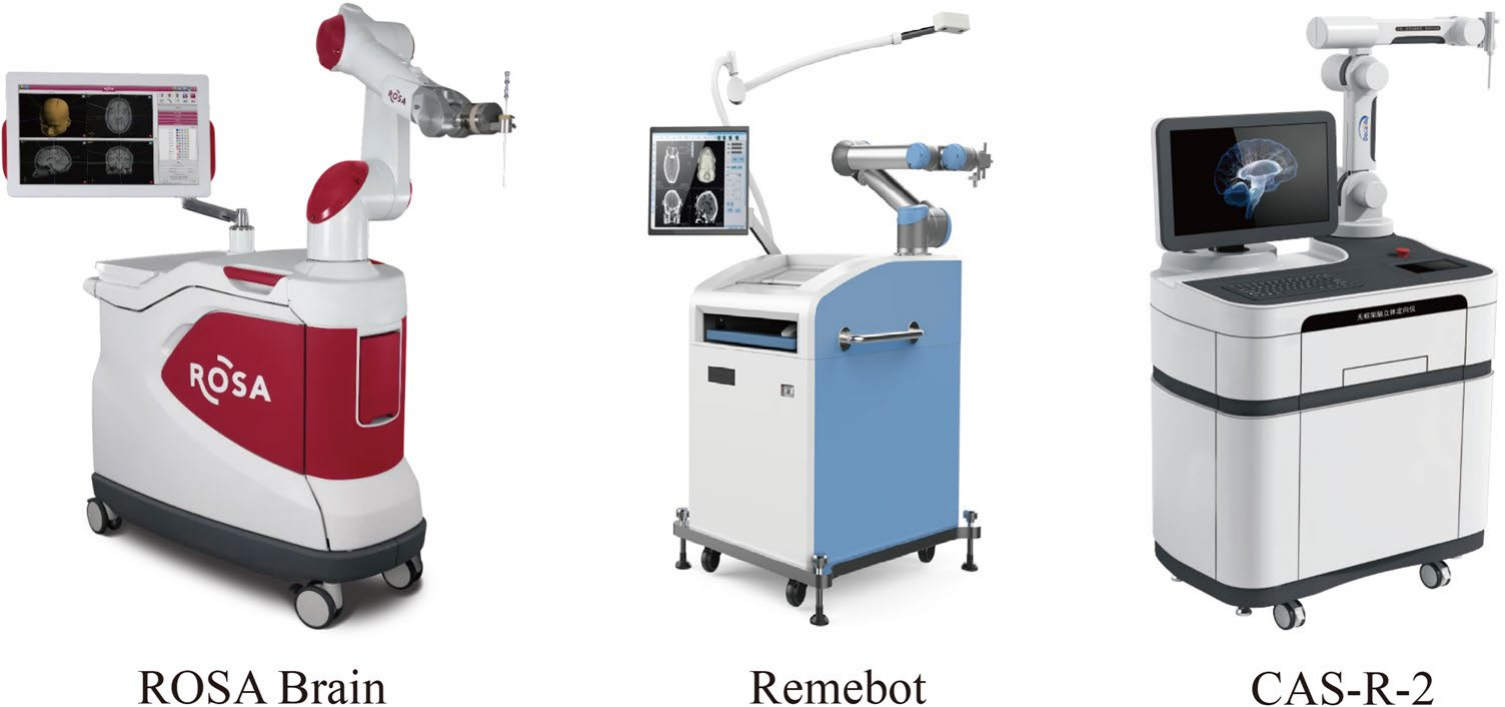
Three commercially available robot system for neurosurgery

The neurosurgical robots can increase accuracy and precision of targeting lesions, provide stable surgical platforms, and make telemedicine a reality [12], which is widely used for neurosurgical procedures such as stereo-electroencephalography, deep brain stimulation and stereotactic biopsy [13]. Currently, several representative surgical robots (Fig. 1) (Table 1), including ROSA^®^ (Zimmer Biomet, Warsaw, Indiana, USA), Remebot^®^ (Remebot Technology Co., Beijing, China), and CAS-R-2 frameless stereotactic system (HOZ Medical Co, Beijing, China), have undergone preliminary validation through small-scale clinical trials for the treatment of ICH. These robots are all commercially available robotic, which employ robotic arms with multiple degrees of freedom to achieve accurate position and integrate the functions of surgical path planning, navigation and control software [14]. The related clinical researches have showed that robotic-assisted surgery for ICH is safe and efficient [15–20].

**Table 1:** The features of ROSA^®^, Remebot^®^ and CAS-R-2 robots

Project	Country	Features	References
ROSA^®^	America	(a) 6-DoF serial robot (b) Multiple registration (c) Force torque sensor technology	[15]
Remebot^®^	China	(a) 6-DoF serial robot (b) Frameless registration (c) Integrated planning software (d)Accuracy: ≤ 0.5 mm	[19]
CAS-R-2	China	(a) 5-DoF serial robot (b) Frameless registration (c) Integrated planning software	[22]

In the general surgical procedure, the preoperative three-dimensional (3D) image is necessary for delineating the relevant anatomy which can be achieved via a CT scan, MRI, or a combination of these images fused together. Then the surgeon determines a desired trajectory based on the central point of the maximum plane and the long axis of the hematoma. After completing registration, the robotic arm automatically moves the target area along the preplanned trajectory. A burr hole is then created as the operation channel, and the surgeon inserts the surgical instruments and completes the operation with the robot’s assistance. After the operation, postoperative imaging is usually used to evaluate the surgical result.

## Key Technologies of Robot-Assisted Surgery for ICH

The integration of robotic systems into the treatment of ICH presents both opportunities and challenges to the surgeon. The following is an overview of several key technologies of robot-assisted surgery for ICH, including stereotactic technique and navigation, the puncture instrument, and hematoma evacuation.

## Stereotactic Technique and Navigation

Robotic devices were first applied to stereotaxy in 1985 when Kuoh combined a PUMA robot and framed stereo locator for intracranial needle biopsy [21]. Subsequently, ROSA^®^, Remebot^®^ and a series of neurosurgical robotic systems were developed. Registration is one of the core of the entire operation, which aligns the preoperative 3D imaging and planned trajectory with the actual patient position in the operating room. There are a variety of different ways to achieve registration, including mechanical based surface registration using facial features or bone fiducial registration. The use of frame-based or bone fiducial-based registration is considered more accuracy to be more accurate for aligning preoperative imaging and intraoperative position, but it may also increases the suffering of patients [22]. The technique entails perforating the patient's skull and affixing metal frame to the bone, both of which can elicit intense discomfort and pain, thus accounting for the heightened suffering experienced by patients.

Currently, computer-aided surgical navigation is widely used in neurosurgery and has achieved good outcomes in the treatment of ICH, with a registration accuracy of 2.12–2.51 mm [23]. However, the current 3D spatial reconstruction and localization methods of ICH are mostly limited to the segmentation and reconstruction of hematoma and brain tissue, and the estimation of possible risk factors (functional areas and vascular distribution) during puncture is insufficient. Traditional surgical navigation systems can realize the localization of the lesion, surgical planning and surgical tool tracking but cannot reflect changes in brain tissue and hematoma in real time.

Intraoperative imaging is one of the solutions and mainly includes intraoperative CT and MRI. Intraoperative CT can quickly and accurately determine the location, volume and morphology of hematoma [24], which can improve the neurological function of patients [25]. However, there are several problems related to intraoperative CT, such as radiation exposure, operation interruption during the scan, and metal artifact interference, and it cannot reflect the relative positional relationship between the hematoma and needle in real time [26]. Intraoperative MRI localization can accurately locate the location, volume and shape of the lesions without radiation. However, there are several disadvantages in using intraoperative MRI for ICH surgery, such as the long imaging time, narrow operating space, the special material requirement for the surgical instruments to adapt to the strong magnetic field, and the contradiction between the magnetic field strength of MRI equipment and imaging quality [27].

Endoscopic technology enables doctors to observe the relative positional relationship between the hematoma and surrounding brain tissue directly from the inside by neuroendoscopy [28]. The advantages are the wide vision of the operative field and the relatively small damage to the brain tissue surrounding the hematoma. Compared with traditional treatment, it can shorten the time of external ventricular drainage and reduce surgical complications [29]. However, the commonly used neuroendoscope lacks a stereo sense, the volume of the lens is too large, and there are problems such as lens atomization and pollution during the operation [30].

Furthermore, augmented reality and mixed reality technologies have been applied to surgical navigation processes, which enable users to engage with digital content and interact with holograms in the real world. Zhou et al. developed a multimodel mixed reality navigation system for ICH surgery, which can provide surgeons with direct observations for preoperative surgical planning and intraoperative tracking of surgical tools. The registration errors were 1.03 and 1.94 mm in phantom and clinical experiments, demonstrating the accuracy and effectiveness of the system and mixed reality technology for clinical application [23].

Compared to functional neurosurgery, MIS for ICH does not demand perfect stereotactic accuracy [31]. However, imprecise registration during MIS for ICH can lead to a deviated trajectory and targeting error, resulting in a higher risk of iatrogenic injury to functional regions and blood vessels, as well as rebleeding [32]. Although the current registration accuracy is adequate for MIS for ICH, ensuring precise registration is crucial to minimize these risks. Therefore, there is still a need to improve registration accuracy for minimally invasive evacuation of ICH. Robotic systems are well-equipped to handle spatial information and directives, and can provide multiple options for registration, which mutually correct each other to enhance accuracy [33]. Additionally, to enhance registration accuracy in minimally invasive evacuation of ICH, robotic systems can utilize multiple sensors and detectors to capture diverse information, high-precision image processing technology to obtain precise surgical scene information, machine learning and artificial intelligence algorithms to assist the robot in recognizing elements in the surgical scene, and real-time feedback and correction mechanisms to monitor and adjust position and posture in real time.

## Puncture Instrument

During MIS for ICH, the puncture needle needs to pass through three types of tissues of different stiffnesses, including the skull (solid, rigid), brain tissue (semisolid, flexible) and hematoma (liquid, soft), the stiffness of which can differ by orders of magnitude [34–36]. Frameless stereotactic robots insert puncture instruments into specific targets through straight-line trajectories within the brain, while the mainstream brain puncture needles in clinical practice are straight and rigid, posing a challenge to the avoidance of critical anatomical structures and the efficacy of hematoma evacuation, especially when hematoma is deep or irregular in shape [14]. Continuous robots, particularly concentric tube robots, are capable of nonlinear motion, which provides a promising alternative for MIS [37].

In 2013, Burgner et al. presented a simple two-tube, 3-degree-of-freedom (DoF) concentric tube robot for image-guided evacuation of ICH, which was composed of a straight, stiff outer needle and a precurved, superelastic aspiration cannula. The authors completed the structural design, image guidance and the optimization method for the selection of the precurvatures of the aspiration cannulas, which can evacuate 83–92% of the hemorrhage volume that in vitro experiments demonstrate [38, 39]. In 2015, Godage et al. integrated intraoperative image feedback and a concentric tube robot in the evacuation of hematoma in a phantom model, which achieved 85% removal of hematoma without appreciable damage to the surrounding brain tissue [40]. Furthermore, Chen et al. fabricated an MR-conditional steerable needle robot for ICH treatment, which achieved hematoma aspiration with a prebent internal tube guided by MRI [41]. Recently, Yan et al. demonstrated a continuum robot design consisting of a precurved cannula and 2-DoF flexible tips for minimally invasive aspiration of hematoma in ICH, which could achieve follow-the-leader (FTL) motion within 2.5-mm shape deviation and control performance within submillimeter errors [42]. In addition to the above steerable needle robots, Sheng et al. introduced a mesoscale medical robot for neurosurgical intracerebral hemorrhage evacuation (NICHE), which employs shape memory alloy actuators to actuate individual degrees of freedom [43].

Compared with the rigid puncture instrument, a steerable needle robot with higher degrees of freedom can reach the bleeding area more dexterously and maximize the evacuation rate of hematoma. However, all reported steerable needle robotic devices are still in the stage of in vitro experiments with no relevant animal experiments or clinical applications. Some of the reasons for this situation are the limitations in controllability, accuracy and functionality. Specifically, flexible movement at the end of the needle driver input and nonlinear mapping led to a decrease in the controllability, and the dynamic interaction between the flexible piercing needle and variable stiffness environment results in higher requirements for motion accuracy [44]. The existing flexible puncture needles only consider the basic motor performance and lack the multifunctional perception required for ICH treatment [37].

## Hematoma Evacuation

It is generally believed by neurologists that removing the hematoma can reduce the damage to brain tissue. The results of the MISTIE III trial showed that only patients who reduced the hematoma by at least 70% or reduced the residual volume of the hematoma to less than 15 mL achieved neurological improvement within 1 year. However, 58% of patients could not achieve this goal during the study [10]. Currently, the artificial hematoma aspiration and drainage used in clinical practice cannot precisely control the operative parameters, such as the speed of aspiration and drainage and the target volume of hematoma clearance. Removing hematoma too quickly can lead to a rapid drop in intracranial pressure and negative pressure in the hematoma cavity, resulting in “decompression injury” and increasing the risk of rebleeding. Besides, the reduction in the compression effect of the hematoma could also increase the risk of rebleeding, particularly in patients with poor cerebrovascular conditions. The use of thrombolytic agents (e.g., RT-PA, urokinase) is an important aspect of MIS for ICH. A series of studies have shown that thrombolytic agents can improve patient outcomes, but clinical and animal studies have also shown that urokinase and RT-PA may have dose-dependent neurotoxic effects and increase the risk of rebleeding [45, 46]. Therefore, the dosage of liquefaction agent needs to be controlled precisely to achieve the goal of dissolving the hematoma and reducing the toxicity and side effects. Currently, the dosage selection of thrombolytic agents is mainly based on medical images and the clinical experience of the surgeon.

Above all, there is a lack of detailed and quantitative indicators to guide MIS for ICH, which is highly dependent on the experience of doctors in clinical practice. More high-quality clinical research and basic research are needed to promote the standardization and individualization of treatment. Additionally, big data and deep learning make it possible to realize intelligent decisions in the diagnosis and treatment of ICH.

## Discussion and Future Perspectives

Patients with ICH inevitably suffer a very poor prognosis, and no definitive treatments have been developed to improve functional outcome after ICH. Despite its therapeutic potential to be employed in the treatment of ICH, there is currently insufficient evidence in the literature to indicate that the efficacy of surgical hematoma removal is significantly better than that of conservative treatment. Irreversible injury induced by ICH, mechanical trauma to normal brain tissue caused by surgical maneuver, low visibility during surgery, deviation of the catheter, and a larger residual hematoma are important factors in the recovery of postoperative nervous function, which are in turn restricted to the current MIS techniques and equipment [10, 47, 48]. Robot-assisted surgery is one of the cutting-edge developments in the field of MIS and has become preferable to traditional minimally invasive modalities, which is crucial for modifying MIS for ICH patients.

However, there are still disadvantages within current surgical robots, which are reflected in the following three aspects. First, craniopuncture needles used for hematoma evacuation have a single function and, thus, may be difficult to apply to a variety of brain tissues of different stiffness, which poses a risk for iatrogenic damage during surgery. Second, utilizing robot-assisted surgery facilitates intraoperative neuronavigation positioning, but puncture remains to be performed manually. Moreover, the robots fail to achieve dynamic real-time monitoring of the puncture process. Therefore, it is difficult to ensure the safety and accuracy of the puncture. Finally, there is a lack of effective means to perceive the intracranial environment, which makes it difficult to remove as many hematomas as possible. Furthermore, there is a paucity of data to quantitatively describe the relationship between the parameter of aspiration procedure and the postoperative rehabilitation efficacy, so it is difficult to control the optimal parameter based on individual factors and lesion characteristics. Collectively, a relatively smooth reduction in the ICP remains elusive.

**Fig. 2:**
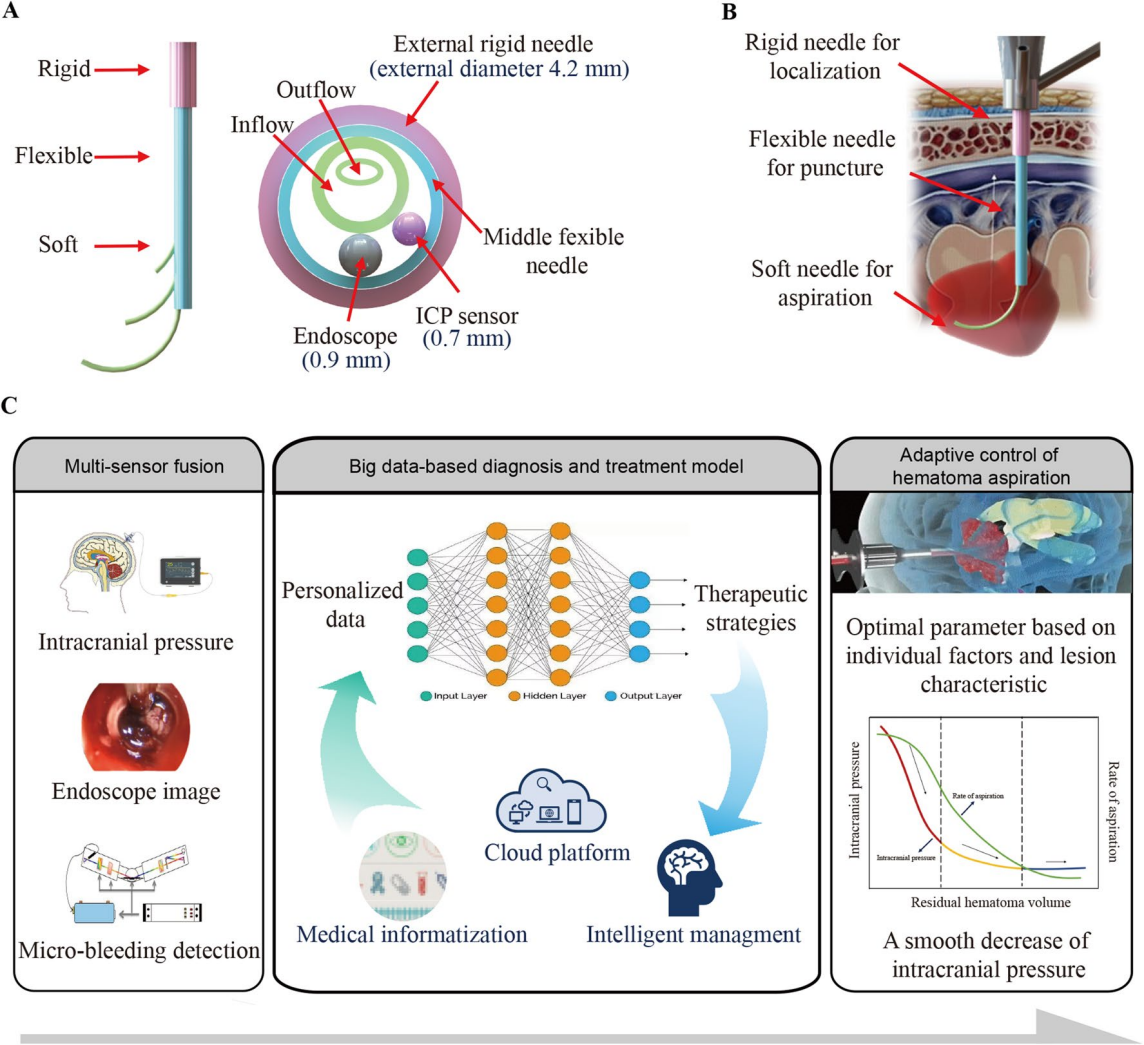
The idea of “Multi-sensor fusion and intelligent aspiration control of minimally invasive surgical robot for ICH”. **A**, **B** show the design of the multi-channel rigid-flexible-soft puncture needle. **C** multi-sensor perception of intracranial environment and intelligent decision-making model for hematoma aspiration

The concept of the Tri-Co Robot (the Coexisting-Cooperative-Cognitive Robot), proposed by Chinese scientists, has emerged as an innovative idea to solve the abovementioned problems. Tri-Co Robot refers to a robot that is able to adapt to dynamic and complex environments autonomously and naturally interact with working environments, humans and other robots [49]. According to this advanced theory, modifying existing surgical robots in aspects such as ontology structure, sensor fusion, and intellectualization is predicted to be capable of facilitating mutual perception between robots and the intracranial environment and finally achieves collaboration between robots and ICH patients as well as doctors. Based on this line of thought, we put forward the following scenario, named “Multisensor fusion and intelligent aspiration control of minimally invasive surgical robot for ICH”, the salient points of which are provided below [50, 51]:

1. Multichannel puncture needle of variable stiffness design: surgical robots with multichannel configuration have been preliminarily explored. Hendrick and colleagues developed a multichannel robotic system for transurethral procedures by adapting a rigid endoscope, which includes two manipulators, two fiber optic bundles, and an endoscope lens, all integrated into an 8.35 mm inner diameter sheath [52]. Furthermore, Yu et al. introduced a concentric tube robotic system featuring three channels, comprising a pair of manipulation channels and a vision channel, all encased within a 10 mm active sheath. The practicality of utilizing the multichannel system for transnasal nasopharyngeal carcinoma procedures was investigated through a range of simulations and experiments [53]. As for surgical robots for ICH, the puncture needle should be available in multichannel design that cover functions such as visualization, perception, and aspiration, which is also highly integrated to reduce iatrogenic injury. Moreover, the development of a rigid-flexible-soft puncture needle is dependent on cannula design to accommodate changes in tissue stiffness (skull, brain tissue and hematoma) and shows adequate performance for motion (Fig. 2A, B).
2. Real-time visualization of the puncture process and autoregulatory control: multi-modal visualization technology, including CT, MRI, ultrasound, and other imaging modalities, has already been implemented in robotic procedures to facilitate intraoperative decision-making. In addition, emerging visualization technologies, such as photodynamic capture and enhanced microscopy, will allow real-time tissue imaging during surgical interventions [54]. In the scenario for surgical robots for ICH, real-time registration of endoscopy images and preoperative imaging profiles (CT and MRI) provides intraoperative visualization of the size, morphology and location of the hematoma and helps to avoid damage to blood vessels. Moreover, during manual surgery, surgeons usually rely on visual or force feedback to adjust their movements, which may not always result in accurate outcomes. However, the development and implementation of adaptive control techniques have enabled the suppression of tremors and improvement of targeting accuracy during robot-assisted surgery. For example, Ebrahimi et al. demonstrated that the use of adaptive control techniques can significantly enhance the safety of sclera force during robot-assisted eye surgery [55]. Therefore, it is recommended to employ adaptive control methods in robot-assisted surgeries for ICH to compensate for real-time puncture errors and achieve autoregulatory control of the puncture process.
3. Multisensor perception of the intracranial environment and intelligent decision-making model for hematoma aspiration: constructing a big data-based innovative diagnosis and treatment decision-making support model leveraging intracranial multimode dynamic perception information, including ICP, detection of microbleeding, and endoscopy images, assisting personalized hematoma evacuation strategies and enabling a smooth decrease in ICP (Fig. 2C).

**Fig. 3:**
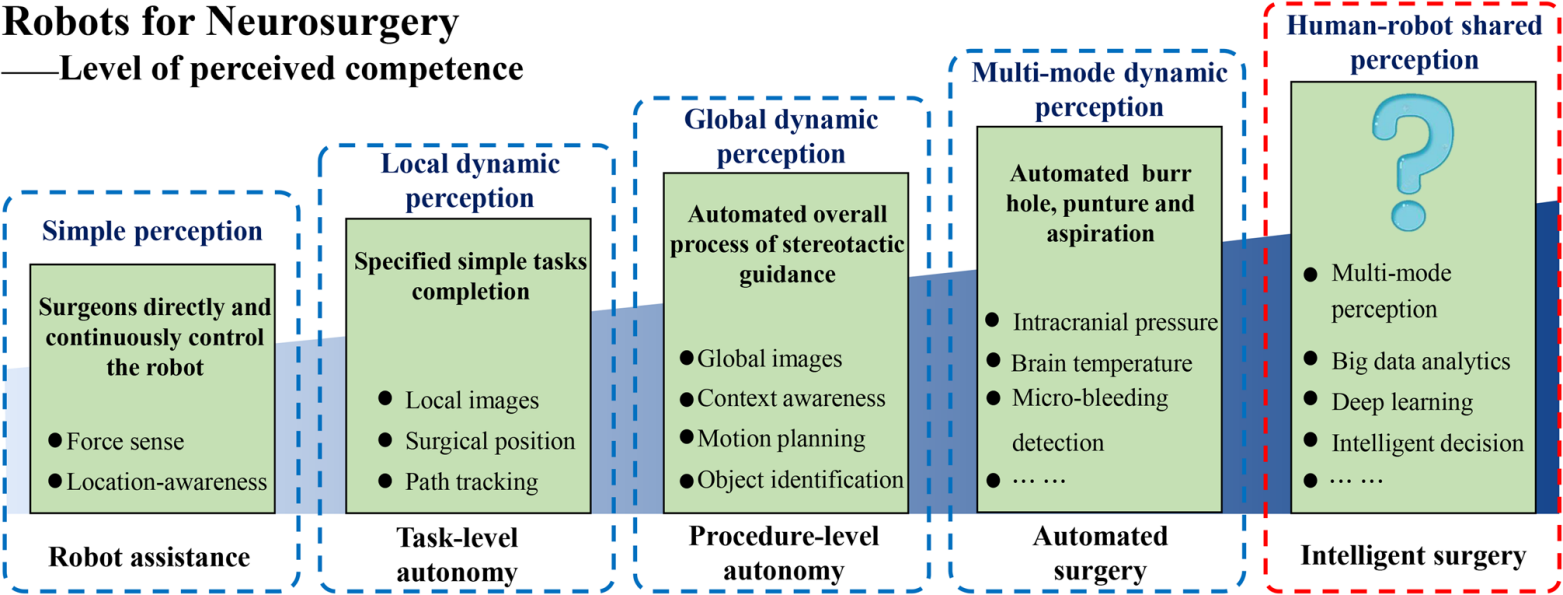
The proposed 5-stage classification for assessing the perceived competence of surgical robots, which correspond to the need of different degrees of autonomy for robot-assisted MIS for ICH

Above all, this scenario is characterized by intraoperative visualization, personalized hematoma aspiration and precise treatment. “Perception” is, of course, a centrally important technique for realizing such a scenario. Actually, perceived competence of surgical robots can be structured into 4 different tiers: simple perception, local dynamic perception, global dynamic perception, and multimode dynamic perception, which correspond to the fulfilment of robot assistance of the surgery, task-level autonomy, procedure-level autonomy and automated surgery (Fig. 3). With the development of robotic and artificial intelligence technologies, we envisage that "human-robot shared perception" is expected to become a higher level of perceived mode. This concept highlights the transformation of abstract clinical experiences into regular patterns through deep learning methods in large datasets, which are then combined with multimode dynamic perception to achieve the precise medical treatment of ICH.

In summary, the new generation of surgical robots for ICH should be subject to big data-oriented decision-making and could participate in the whole procedure of hematoma puncture and aspiration, finally revolutionizing MIS and facilitating quantitative, precise, individualized, standardized treatment strategies for ICH.
